# The Influence of Perceived Red Tape on Public Employees’ Procrastination: The Conservation of Resource Theory Perspective

**DOI:** 10.3390/ijerph19074368

**Published:** 2022-04-05

**Authors:** Qiufeng Huang, Kaili Zhang, Ali Ahmad Bodla, Yanqun Wang

**Affiliations:** 1 School of Political Science and Public Administration, Huaqiao University, Quanzhou 362021, China; hqf1988@hqu.edu.cn (Q.H.); m13547893885@163.com (Y.W.); 2 School of Business, East China University of Science and Technology, Shanghai 200237, China; 3 Management School of Hainan University, Hainan University, Haikou 570228, China; alibodla22@gmail.com

**Keywords:** procrastination, perceived red tape, role overload, perceived overqualification, conservation of resource

## Abstract

Procrastination is a prevalent phenomenon in organizations, yet limited knowledge is available on how situational antecedents influence it. Based on the conservation of resource theory, we explore how and when perceived red tape influences public sector employees’ procrastination behavior. Using survey data of 751 public sector employees from China, we revealed that perceived red tape is positively associated with procrastination behavior, and role overload partially mediates the relationship between perceived red tape and procrastination behavior. Employees’ perceived overqualification augments the relationship between role overload and procrastination. Further, the moderated mediation model test illuminates that the indirect effect of perceived red tape on procrastination through role overload depends on perceived overqualification, which means that higher perceived overqualification amplifies the indirect effect. Our research enriches the literature on public sector employees’ procrastination behavior.

## 1. Introduction

Procrastination, generally observed in individuals who consciously avoid work and indulge in irrelevant actions while completing their assigned work [1,2,3,4], has become a prevalent phenomenon at the workplace [3,5]. According to surveys, 30–65% of the staff browse websites or engage in activities irrelevant to their work, even when faced with urgent and essential tasks, such as project deadlines [5]. Procrastination has been found detrimental to individuals’ performance [3,6,7,8]. The extant research studies on procrastination have emphasized mainly concept definition and scale development [9,10,11], while some empirical research focused on the situational antecedents of procrastination. For example, studies have found that procrastination is influenced by job design and job demand. Individuals are highly likely to procrastinate when job design is objectionable or they encounter a heavy workload [3,12].

Although previous studies revealed insightful findings, they overlooked different contexts and contingent factors affecting procrastination of public sector employees [3,10,12]. Scholars have theorized that employees in public sectors have a higher level of perceived red tape than individuals in a private organization because of complex bureaucratic procedures [13,14]. Perceived red tape is characterized as burdensome and as ineffective rules in an organization [15]. The literature on perceived red tape discussed the relationship between perceived red tape and individuals’ attitudes and behavior, such as public service motivation [16], job satisfaction [17], work engagement [18], performance [19], innovation [20], and team involvement [21]. However, to the best of our knowledge, relationships between perceived red tape and public employees’ procrastination have been overlooked. Understanding the relationship between perceived red tape and public employees’ procrastination is vital for public sector management efficiency and performance. Therefore, the primary purpose of this study is to examine the effect of perceived red tape on public employees’ procrastination behavior. In addition, it explores how and when the factors (role overload and perceived overqualification) influence the relationship between perceived red tape and procrastination.

Drawing on the conservation of resource (COR) literature [22], individuals avoid resource loss and obtain additional resources when external situations raise unfavorable threats evaluation [23,24,25]. As procrastination is a behavior taken to avoid work, this study regards procrastination as a self-resource conservation behavior adopted by individuals to avoid potential resource consumption [22,25,26]. According to perceived red tape conceptualization, it can trigger a precautionary psychological process of handling work overload; we posit a positive relationship between perceived red tape and procrastination of public employees. Because of conflicting dual-task requirements of fulfilling the essential public service supply and dealing with the work procedures of perceived red tape, public employees are likely to result in a high level of role overload, which leads to procrastination behavior. Therefore, this study further explores how role overload conduits the relationship between perceived red tape and procrastination.

However, public employees differ in the extent between resource depletion and outcomes [22,27]. Our study expects that perceived overqualification will moderate the indirect relationship between perceived red tape and procrastination. Perceived overqualification is an individual’s perception that his or her education level, experience, knowledge, and skills are higher than the job requirements [28]. Perceived overqualification is a common issue in the public workplace due to the traditional Chinese culture of “excellence leads to an official” and the high work value of job security [29]. For example, the news reported that fierce competition for street cleaner management positions attract many graduates of master’s and doctoral degrees from prestigious universities because the positions offer permanent job security in the public sector. Individuals’ perceptions of overqualification affects their work attitudes, emotions, and cognition [30,31]. Therefore, this study further examines the boundary effect of perceived overqualification on the relationship between perceived red tape and procrastination.

This study makes several contributions to the public administration literature. First, we extend the literature on procrastination behavior by clarifying the effect of perceived red tape on public employees’ procrastination. Second, we highlight the role overload as the central psychological mechanism in the relationship between perceived red tape and procrastination based on the conservation of resource theory perspective. It broadens our understanding of how a psychological factor of perceived red tape leads to individuals’ procrastination. Finally, we also provide empirical evidence of the moderating mechanisms of perceived overqualification underlying the relationship between perceived red tape and public employees’ procrastination. In practice, our research should benefit public organizations by providing suggestions for improving public employees’ administrative efficiency.

## 2. Theoretical Development

### 2.1. Procrastination as Resource Conservation Behavior

Procrastination refers to an individual’s conscious behavior to engage in relaxing activities and to avoid completing tasks [1,2,3,4]. Based on this conceptualization, procrastination at the workplace is characterized by employees’ intentional actions to avoid assigned work. Individuals display procrastination by indulging in relaxing activities unrelated to work when assigned to perform specific tasks. Procrastination can result in adverse outcomes for employees, such as a tight work schedule and a high emotional burden [3,8].

Existing research on procrastination has focused mainly on procrastination in non-workplaces, such as students’ procrastination in their study assignments and individuals’ procrastination in time management of their daily lives [5,11,32,33]. Due to a fragmented understanding of workplace procrastination, researchers have mainly discussed the concept and dimensions of procrastination. For instance, some studies have conceptualized procrastination as a two-dimensional concept. One dimension of procrastination is exhibited through task avoidance and participation in activities irrelevant to work, such as daydreaming and relaxing. The second refers to using internet applications by employees who pretend to work in front of the computer while shopping online, browsing websites and social media, and reading gossip [1,2,32]. Although it is possible to divide the two types of procrastination theoretically, they are challenging to differentiate in practice. Therefore, research on procrastination has treated it as employees’ overall behavioral expression of delaying work without emphasizing the different dimensions. Following the mainstream about procrastination behavior, this study treats procrastination as individuals’ passive workplace behavior without emphasizing its different dimensions.

Procrastination is characterized by individuals engaging in short-term delaying behavior to avoid completing the work on hand. Usually, individuals tend to procrastinate because they consider that performing the task can consume ample resources; therefore, they procrastinate to avoid potential resource consumption. In this situation, the more complex the task is, the more likely individuals will procrastinate the work [1,2,3,4]. Procrastination is manifested in short-term leisure and relaxation behavior, which can be a tactic for preserving self-resources [22,27]. Based on the above discussion, procrastination prevents potential resource consumption and preserves current resources, which are the main arguments in the COR literature [25,26]. Therefore, this study views procrastination as an individual’s resource conservation behavior and explains why individuals procrastinate in the public sector.

### 2.2. Perceived Red Tape and Procrastination

Perceived red tape refers to rules, regulations, and procedures that remain in force and entail a compliance burden but do not advance the legitimate purpose that the rules were intended to serve [15]. Red tape is characterized as burdensome, unnecessary, and ineffective organizational policies and procedures [34]. Most recently, studies have proposed that the detrimental impact of perceived red tape on employee outcomes is based on the job demands–resources model, considering the perceived red tape as one of a hindering job demands [16,35]. Extending the COR theory to the perceived red tape literature, we argue that perceived red tape directly impacts individuals’ resource threat evaluations [23], which may engender public employees’ resource conservation behavior to protect their current resources and to avoid further resource loss.

High-level perceived red tape presents a more considerable compliance burden of the rules that public employees have to comply with, and it requires individuals to expend more energy, time, and psychological resources to utilize. Research has shown that perceived red tape is negatively associated with job satisfaction [17] and job involvement [16], as well as positively related to stress [36] and burnout [37]. Furthermore, public employees with high red tape perception will fail to obtain a sense of self-efficacy and job meaning because the rule’s compliance burden has no legitimate goal [15]. Additionally, the essence of red tape is the failure and distortion of work rules and procedures, which is one of a hindering job demand [16,35]. As an unreasonable and ineffective job demand, it is apt to form the tendencies of red-tapism and formalism in the work practice, which undermine public employees’ sense of control and self-determination in administrative work [38,39,40,41]. Thus, public employees believe that any effort to work under high perceived red tape would only lead to resource consumption with no legitimate goal, and then they are more likely to take actions to protect their current resources and to avoid further resource loss. Since we consider procrastination as employees’ resource protection behavior, we thus hypothesize that perceived red tape would lead to public employees’ procrastination behavior. Based on the above discussion, we posit:

**Hypothesis** **1** **(H1).** *Perceived red tape is positively related to public employees’ procrastination*.

### 2.3. The Mediating Effect of Role Overload

Role overload refers to the perception of role conflict when individuals perceive themselves to undertake too much responsibility and expectation beyond their available time and energy [42]. Red tape indicates a deviation when organizations strive for the balanced development of organizational legitimacy goals and benefit goals [29,43]. The complex procedures and rules of red tape facilitate pursuing organizational goals of adapting the institutional environment and legitimizing organizational functions. However, it inhibits the management efficiency goal of the organization. Public employees invest excessive time and energy but fail to reach the organizational efficiency goal. Therefore, public employees tend to “go through the motions faithfully” in actual work [28], which exerts extra work. In brief, public employees who face red tape such as formalistic conferences, excessive documentation, and reporting useless data, reports, and materials will perceive a role of conflict and stress because they are inevitable under the requirements of the conflicting dual tasks of fulfilling the essential public service supply and dealing with the red tape [44]. Empirical studies have also shown that perceived red tape leads to a high role conflict perception and work stress [45]. Therefore, the hypothesis is proposed:

**Hypothesis** **2** **(H2).** *Perceived red tape is positively associated with role overload*.

Role overload indicates that public employees consume significant psychological resources, leading to the resource threat evaluation [42]. The conservation of resource theory proposes that individuals are more likely to take passive actions to preserve self-resources due to the resource loss evaluation [25,46]. When individuals encounter many tasks unrelated to the actual public service work by the red tape procedures, it consumes psychological resources (e.g., employees perceived role pressure, emotion anxiety, tension, and restlessness) [47]. Therefore, individuals hope to take resource conservation behaviors to protect their current resources and to avoid further resource loss [25,46]. Empirical studies have shown that a high workload can directly impact individual resource consumption and resource threat, resulting in negative perception and job avoidance [8,12,42]. Therefore, the hypothesis is proposed:

**Hypothesis** **3** **(H3).** *Role overload is positively associated with public employees’ procrastination*.

In conclusion, based on the conservation of resource theory, public employees are likely to generate high levels of role overload perception in the face of ineffective, burdensome, and unavoidable work tasks from perceived red tape. These overloaded role pressures and conflicts would significantly erode their internal psychological and emotional resources and make them feel exhausted mentally and physically. In this condition, public employees tend to take corresponding resource conservation behaviors to supplement their inner psychological resources in their work, resulting in more procrastination behaviors. Therefore, the hypothesis is proposed:

**Hypothesis** **4** **(H4).** *Public employees’ role overload mediates the relationship between perceived red tape and procrastination*.

### 2.4. The Moderating Effect of Perceived Overqualification

Perceived overqualification is a subjective assessment of being overqualified and underemployed due to a mismatch between an individual’s skill levels (education, experience, knowledge) and the job [28]. Public employees with high perceived overqualification are prone to produce feelings of relative deprivation. They are afraid of breaking the psychological contract of mutual benefit between individuals and organizational units because of their higher expectations about the job [30,31]. When high perceived overqualified public employees face higher levels of role overload, they will perceive a greater sense of psychological resources shortage and are more likely to feel demotivated at work. In addition, with high perceived overqualification, public employees usually have more negative psychological affection such as frustration, disappointment, and anger at work [29,30]. They, thus, are more likely to perceive resources threat and may be less willing to put forth their effort when completing the duties. Conversely, if low perceived overqualified public employees (i.e., those expected to experience less negative attitudes and emotions in the person–job mismatch) are under the role overload, they are less likely to perceive more resources threats. Thus, we expect that high perceived overqualification under role overload will generate more procrastination than those low overqualification public employees. Therefore, the hypothesis is proposed:

**Hypothesis** **5** **(H5).** *Perceived overqualification moderates the positive relationship between role overload and procrastination in such a way that the positive relationship between role load and procrastination is strengthened under high perceived overqualification*.

Based on the conservation of resource theory, perceived red tape led public employees to a higher level of role overload, generating a sense of resources threat. Public employees tend to reduce job involvement and adopt more negative coping strategies such as procrastination to prevent the further loss of psychological resources. The influence of perceived red tape on procrastination is mediated by role overload. The indirect influence is affected by perceived overqualification. A high level of perceived overqualified public employees generates negative attitudes and emotions, further aggravating their sense of depletion of psychological resources and strengthening the indirect effect of perceived red tape on procrastination through role overload. Therefore, the moderated mediation model hypothesis is proposed:

**Hypothesis** **6** **(H6).** *Perceived overqualification moderates the indirect effect of perceived red tape on procrastination through role overload such that the indirect effect is stronger when perceived overqualification is higher*.

**Figure 1:**
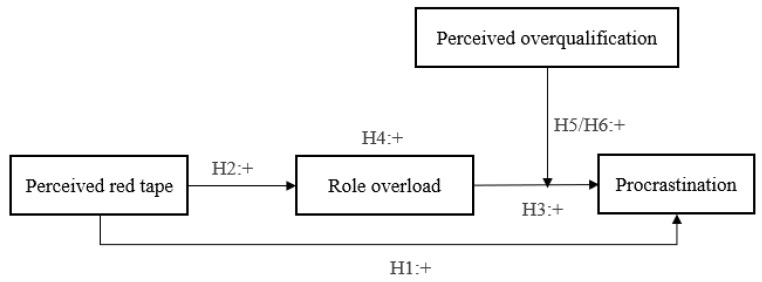
The theoretical framework of the study.

Based on the above analysis, the theoretical model figure of this study is shown in Figure 1 below.

## 3. Method

### 3.1. Sample and Procedures

The current study sample was composed of public sector employees of China. After obtaining approval from the university ethics committee, we collected data via Wenjuanxing online platform. A two-wave data collection was conducted in the current study. In the first stage (Time 1), the participants filled out their demographic information, perceived red tape, role overload, and perceived overqualification. Demographic information included gender, tenure, educational level, marriage status, the administrative rank of their organization, officer position, job position, type of work. The Time 1 survey included 869 participants. In the second stage (T2, conducted one months later), the participants were asked to assess their procrastination. We assured the participants that the research survey was only for academic purposes, and there were no right or wrong answers. We provided participants with a consent form and assured them of data confidentiality and anonymity. Participation in the study was voluntary, and participants were free to stop responding to the survey questionnaire anytime. Based on the participants’ e-mail, the research assistants paired the two waves of responses. Seven hundred fifty-one valid questionnaires were received, and the overall response rate was 86.4%. Of the responses, 54.5% were male, and 63.4% were married. In addition, 89.9% of the subjects were graduates with a bachelor’s degree. Of the subjects, 83.9% were at the deputy level or below. However, 35.3% of the participants were from administrative units at the municipal level, and 35.4% were from administrative units at the county and district levels. Of the participants, 54.7% had less than seven years’ tenure, and 43% had tenure of over seven years. The average age of the participants was 32.51 (SD = 10.65).

### 3.2. Measures

The survey items were adopted from the English-language scale. The survey instrument was administered in Chinese; therefore, all items underwent a standard back-translation process [48]. All items were first translated into Chinese by a management professor and then back-translated into English independently by another management scholar. Further, one bilingual management professor compared the two versions of the scales and made modifications to resolve the discrepancies. All measures were rated on a scale ranging from 1 (“strongly disagree”) to 5 (“strongly agree”).

Perceived red tape. We measured perceived red tape using three items based on a validated scale developed by Jacobsen et al. [49]. Sample items of perceived red tape were “rules and procedures make work processes in the organization more troublesome than they need to be” and “the rules and procedures I must follow in carrying out primary work tasks are very time-consuming”. The reliability coefficient of perceived red tape was 0.81. Factor analysis indicated three items loaded on a single factor, and the factor loading score ranged from 0.72 to 0.83 (AVE = 0.61).

Perceived overqualification. Perceived overqualification was assessed using a nine-item scale developed by Maynard et al. [50]. A sample item of perceived overqualification was “My job requires less education than I have”. This scale measured individuals’ perceptions of surplus education, experience, and KSAs (knowledge, skills, and abilities) relative to job requirements. The reliability coefficient of perceived overqualification was 0.89. Factor analysis indicated that nine items were loaded on a single factor, and the factor loading score was ranged from 0.63 to 0.77 (AVE = 0.50).

Role overload. We assessed role overload using the three-item scale adopted from Schaubroeck et al. [51] and Beehr et al. [52]. Sample items of role overload were “I have too much work to do everything well” and “I never seem to have enough time to get everything done”. The reliability coefficient of role overload was 0.88. Factor analysis indicated the three items loaded on a single factor, and the factor loading score ranged from 0.82 to 0.88 (AVE = 0.72).

Procrastination. Procrastination was assessed using an eight-item scale developed by Tuckman [53]. The items were as follows: I needlessly delay finishing jobs, even when they are important; I manage to find an excuse for not doing something; I am an incurable time-waster; I am a time-waster now, but I cannot seem to do anything about it; I promise myself I will do something and then drag my feet; Even though I hate myself if I do not get started, it does not get me going; I get stuck in neutral, even though I know how important it is to get started; Putting something off until tomorrow is now the way I do it. The factor loadings of all individual items ranged from 0.75 to 0.89. The reliability coefficient for procrastination was 0.96 (AVE = 0.74).

Control variables. During the analysis, we controlled for several variables, including gender, tenure, education, marriage status, the administrative rank of their organization, officer position, job position and type of work.

### 3.3. Analytical Strategy

A two-step strategy was applied to test the theoretical model. First, a confirmatory factor analysis (CFA) and descriptive analysis were conducted. CFA was used to confirm the validity and reliability of the measurement model, and the descriptive analysis showed the correlations among the key variables. In the second step, regression analyses were performed to test the hypotheses.

We adopted the method proposed by Baron and Kenney to verify whether role overload mediated the effects of perceived red tape on procrastination [54]. In addition, we conducted the mediation test in which perceived red tape was kept as the predictor, role overload as the mediator, and procrastination as the dependent variable by the method of Preacher and Hayes [55]. We also performed a hierarchical regression analysis for testing the moderating effect of perceived overqualification. We entered all the control variables in Step 1. Then, we entered the independent variables, including perceived red tape and perceived overqualification, in Step 2. Lastly, we entered the interaction terms (role overload × perceived overqualification) in Step 3. Finally, we test moderated the mediation of hypothesis 6 by the PROCESS program [56].

## 4. Results

### 4.1. Preliminary Analysis

We performed Harman’s one-factor test using unrotated principal-component factor analysis to show the common method variance [57]. The analysis extracted four factors with eigenvalues greater than 1. The first factor accounted only for 29.7% of the variance. We interpret the absence of a single factor that accounts for most of the variance as evidence that common method variance poses no serious problem.

We conducted a series of CFAs to examine the construct validity of the multi-item variables in our study using Mplus 7.2 (Muthén & Muthén, Los Angeles, CA, USA). The CFA results are presented in Table 1. We tested the hypothesized four-factor model by loading items on their latent variables. The results revealed that the hypothesized four-factor model fit the data best with ***χ^2^*** = 1026.76, df = 224, root mean square error of approximation (RMSEA) =.069, confirmatory fit index (CFI) = 0.97, normed fit index (NFI) = 0.97, standardized root mean square residual (SRMR) = 0.051. The three-factor model was constructed by combining perceived red tape and perceived overqualification (***χ^2^***= 1729.02, df = 227, RMSEA = 0.094, CFI = 0.95, NFI = 0.94, SRMR = 0.067), which yielded a worse fit than the four-factor model (Δ***χ^2^*** = 702.26 (Δdf = 3), *p* < 0.01). All other alternative factor models fit significantly worse than the proposed model. Hence, the CFA results support the four-factor model.

Table 2 presents the means, standard deviations, and correlations of these variables. The results showed that perceived red tape was positively correlated with procrastination (r =.341, *p* < 0.01); role overload was positively correlated with procrastination (r = 0.250, *p* < 0.01); perceived red tape was positively correlated with role overload (r = 0.460, *p* < 0.01); perceived overqualification was positively correlated with procrastination (r = 0.457, *p* < 0.01). These results provide initial support for our hypotheses.

### 4.2. Tests of Hypotheses

Hypothesis 1 predicted that perceived red tape has a positive effect on procrastination. As expected, Model 4 in Table 3 indicates that perceived red tape was positively related to procrastination (γ = 0.451, *p* < 0.01), Hence, Hypothesis 1 was supported. The results, Model 2, show that perceived red tape was positively related to role overload (γ = 0.446, *p* < 0.01); Hypothesis 2 was supported. Hypothesis 3 predicted that role overload has significant effects on procrastination. Model 5 in Table 3 indicates that role overload was positively related to procrastination (γ = 0.382, *p* < 0.01). Thus, Hypothesis 3 was supported.

Hypothesis 4 predicted that perceived red tape has significant indirect effects on procrastination through role overload. We entered the role overload in model 5 in Table 3. The results show that the positive relationship between perceived red tape and procrastination became weak (γ = 0.382, *p* < 0.01) compared to model 4. The results provided evidence for partial mediation effects of role overload. In addition, we conducted mediation analyses using the method developed by Preacher and Hayes [55]. Preacher and Hayes suggested using a bootstrap method to compute a confidence interval around the indirect effect (the path through the mediator) [55]. If zero falls outside of this confidence interval, the mediation is present. We estimated a 95% confidence interval via 5000 bootstrap re-samples with perceived red tape as our independent variable, procrastination as our dependent variable, and role overload as our mediating variable. The results showed that the indirect effect was 0.070, SE = 0.026, at a 95% confidence interval, the indirect effects were (0.019, 0.122), not containing zero [58]. Hence, Hypothesis 4 was supported.

**Figure 2:**
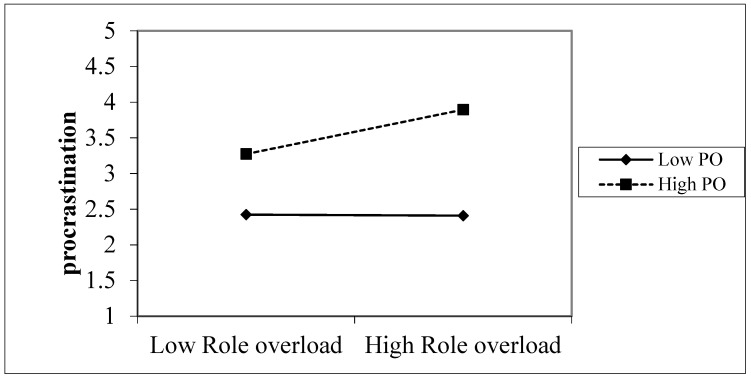
Interactive effect of role overload and perceived overqualification on procrastination.

Hypothesis 5 predicted that perceived overqualification moderates the relationship between role overload and procrastination. When testing moderation effects, we centralized the variables. Model 3 in Table 4 indicates that the interaction term of perceived overqualification and role overload was positively related to procrastination (γ = 0.159, *p* < 0.01). Simple slope analyses indicated that role overload was positively related to procrastination (γ = 0.242, *p* < 0.01) with high perceived overqualification (1 standard deviation above the mean), and this positive relationship became non-significant (γ = −0.38, *p* > 0.05) when individuals’ perceived overqualification was low (one standard deviation below the mean). Hence, Hypothesis 5 was supported, indicating that the positive relationship between role overload and procrastination became more salient under high perceived overqualification. We plot this positively moderating effect in Figure 2.

Following Hayes’ PROCESS macro (Model 14), we tested Hypothesis 6 using the moderated path analysis approach [59,60]. In Hypotheses 6, we proposed that the indirect effect of perceived red tape on procrastination via role overload is more positive when perceived overqualification is high than when perceived overqualification is low. As the results show in Table 5, the conditional indirect effect of perceived red tape on procrastination via role overload was statistically significant when perceived overqualification was high (+1SD, indirect effect = 0.108, 95% CI = (0.040, 0.180)) compared with when perceived overqualification was low (−1SD, indirect effect = −0.17, 95% CI = (−0.065, 0.028)). For additional checks, we consulted the index of moderated mediation, which assesses whether there is a statistically significant difference between two values of the conditional indirect effect at different levels of the moderator [59]. The index of moderated mediation provided supportive evidence for perceived overqualification (indirect effect = 0.070, SE = 0.018, 95%CI = (0.038,0.108)). Hypothesis 6 was supported.

## 5. Discussion

### 5.1. Results and Findings

Drawing on the COR theory, the research focuses on the procrastination behavior of public sector employees and explores the triggering factors of procrastination and its mechanism. Specifically, regarding procrastination as the resources conservation of individuals, the research puts forward that perceived red tape would deplete internal resources cognitively and psychologically of public employees and cause high levels of role overload, thus resulting in more procrastination. In addition, the perceived overqualification of public employees could also amplify the positive indirect relationship between perceived red tape and procrastination via role overload. On account of the data of 751 public sector employees, the results demonstrate a positive relationship between perceived red tape and procrastination behavior and a positive relationship between role overload and procrastination. Furthermore, the role overload partly mediates the effect of perceived red tape on procrastination, and the perceived overqualification of public employees moderates the positive effect of role overload on procrastination behavior. Specifically, the positive relationship between role overload and procrastination behavior is stronger when perceived overqualification is high than when perceived overqualification is low. In addition, the moderated mediation model shows that perceived overqualification moderates the indirect effect of perceived red tape on procrastination through role overload. Thus, the indirect effect is stronger when perceived overqualification is high than when perceived overqualification is low.

### 5.2. Theoretical Implications

There are four following theoretical contributions in this study. First of all, our study is among the frontiers in focusing on the procrastination behavior of public sector employees. While procrastination is widely recognized in the workplace, existing antecedent studies of procrastination emphasize procrastination among employees in the private sector [10]. However, because previous research focuses on the management situation of private enterprises, the conclusion cannot be applied to the management situation of public sectors. The existing research pays little attention to the procrastination behavior of employees in the public sector. In particular, the factors and mechanisms of public employees’ procrastination behavior have been poorly understood [3,8]. In the specific context of the public sector, it is revealed that perceived red tape significantly influences employees’ attitude and behavior [20,40,44,61]. By viewing procrastination as an individual’s resource conservation behavior, the study provides a new idea for understanding public employees’ procrastination behavior and its antecedent factor of perceived red tape in the public sector. Our finding expands the organizational context and antecedent factor in the procrastination literature.

Second, we enrich the empirical evidence of the effect of perceived red tape by demonstrating the positive relation between perceived red tape and the procrastination behavior of public employees. Existing literature on the effect of perceived red tape is ambiguous. Some studies stated that perceived red tape was beneficial; for example, perceived red tape increases organizational change, which is pivotal for organization development [62,63]. Some studies proposed that perceived red tape had an adverse effect. For example, perceived red tape damps the public service motivation of public employees [64], weakens the job satisfaction of civil servants, and reduces their organizational commitment, work engagement, and work performance [34,44]. Other studies showed that perceived red tape had an U-shaped effects. For instance, Lin (2021) confirmed that weak perceived red tape could activate the creative behavior of the public employee, but strong perceived red tape inhibits such behavior [38]. In general, empirical studies on the effects of perceived red tape are not consistent. This study discusses the effect of perceived red tape on public sector employees’ procrastination and confirms the adverse effect of perceived red tape. Moreover, this study also provides micro-evidence support for existing policies to eradicate red tape and reduce burdens for public employees.

Third, this study also proves the underlying mechanism of perceived red tape on procrastination behavior and confirms the mediating effect of role overload. The COR theory argues that individuals’ judgment of external situational factors influences their sense of resource threat and ultimately affects their behavior [65]. Specifically, public employees deal with ineffective and redundant work rules resulting from the red tape; they are more likely to produce a higher workload. These overloaded role requirements usually lead to significant depletion of psychological and physical resources, which would prompt more resource conservation to complete internal resources, leading to more procrastination. Overall, this study proves the mediating effect of role overload on the relationship between perceived red tape and procrastination and expands the application research of the conservation of resource theory in perceived red tape, role overload and procrastination.

Finally, we deepen the academic understanding of the adverse effects of perceived overqualification among public sector employees by revealing its moderating role. Influenced by the Chinese traditional culture of “excellence leads to an official” and the “iron rice bowl” work values make a civil servant position attractive. The phenomenon of high talent consumption is more and more common in public sector management. Existing research mainly focuses on the main effect of perceived overqualification [66,67,68]. Studies have shown that perceived overqualification might have been negatively related to individuals’ attitudes and behavior, such as a psychological contract between individuals and organizations [67], organizational-based self-esteem and career satisfaction [32], psychological well-being and organizational commitment [68,69], and organizational citizenship behavior [31]. This study introduces the moderating effect of perceived overqualification in the path of “perceived red tape—role overload—procrastination” and deepens academic understanding of the adverse effects of perceived overqualification among public sector employees.

### 5.3. Practical Implications

This study has the following important implications for practice. First of all, leaders in public sectors need to pay attention to the detrimental impact of perceived red tape. Public employees produce role conflict evaluations between perceived red tape and practical public service supply constricted due to limited energy and time. Thus, more inappropriate behaviors happen, such as procrastination.

Second, leaders in public sectors should actively pay attention to the levels of role overload of public employees. Multiple role pressure and conflicts from systems, superior departments, or public opinion are the critical factors influencing the public employees’ working attitude and behavior. For example, this study shows that perceived red tape promotes role overload of public employees, leading to more procrastination. Therefore, leaders in public sectors should firmly carry out the policy of reducing burdens for public employees. Public sectors need to eradicate formalism, red-tapism, and excessive procedures and rules for public employees to save valuable energy and time to improve public service effectiveness.

Finally, when faced with perceived red tape, public employees with a high perceived overqualification have a stronger sense of depletion of psychological resources, resulting in more procrastination. Therefore, when recruiting public employees, public departments should combine the specific position competence and stick to the principle of “tapping the full potential of talents” to avoid the phenomenon of talents being perceived as overqualified for their jobs. In addition, in the way of work, superior leaders set challenging tasks such that public employees can reduce their perceived overqualification, stimulate their work engagement, and restrain their negative coping behaviors such as procrastination.

### 5.4. Research Limitations and Future Directions

This study had the following limitations. First, although internal resource storage can have a powerful impact on public employees’ behavior, the importance of workplace support as a resource supply for helping public employees deal with the red tape cannot be ignored. It is suggested that future studies explore the role of organizational and collegial support in influencing public employees’ procrastination behavior [22]. Second, this study found a partial mediating effect of role load between perceived red tape and public employees’ procrastination, and future studies could explore the other underlying mechanism. For instance, based on cognition–affection–behavior [70], public employees with a high level of perceived red tape are more likely to have negative affection, which leads to more procrastination behavior. Therefore, future studies could further explore the other underlying mechanisms linking perceived red tape and public employees’ procrastination behavior. Third, this study focused on the antecedents of procrastination; however, the outcomes of procrastination should also be further explored. While most studies mention that procrastination is harmful, as it leads to anxiety and adversely affects performance, our knowledge of procrastination outcomes remains limited. In addition, some studies have mentioned that procrastination could be a time management strategy, as employees procrastinate to stimulate their efficiency when facing work deadlines [71]. However, empirical research is still limited in this regard. Further studies should examine whether procrastination can make employees more productive when facing deadlines. Fourth, we avoid the common method issue by collecting data at two different time points. However, this method cannot draw the internal causality conclusion. We suggest that further studies adopt an experimental design to show the internal causality.

## 6. Conclusions

Based on the conservation of resource theory, the research provided a theoretical model of perceived red tape, role overload, perceived overqualification, and public employee procrastination. The research findings revealed that perceived red tape is positively associated with procrastination behavior of public employees, and role overload partially mediates the relationship between perceived red tape and procrastination behavior. Perceived overqualification of public employees strengthens the positive relationship between role overload and procrastination. In addition, the moderated mediation model test demonstrated that the indirect effect of perceived red tape on procrastination through role overload depends on the degree of perceived overqualification, which means that higher perceived overqualification amplifies the indirect effect. The research enriches the theoretical research on procrastination behavior among public sector employees. Furthermore, it broadens knowledge that organizations can dilute public employees’ procrastination behavior by managing the workload and person-job alignment. It would facilitate the public employees’ efficiency and meaningfulness at work.

## Figures and Tables

**Table 1:** Confirmatory factor analysis.

Model	*χ^2^*	*df*	Δ*χ^2^*(Δ*df*)	RMSEA	CFI	NFI	SRMR
Four-factor model (Hypothesized model)	1026.76	224	----	0.069	0.97	0.97	0.051
Three-factor model A	1729.02	227	702.26 ** (3)	0.094	0.95	0.94	0.067
Three-factor model B	2202.00	227	1175.24 ** (3)	0.110	0.93	0.92	0.082
Three-factor model C	2514.08	227	1487.32 **(3)	0.116	0.92	0.92	0.110
Two-factor model D	3190.82	229	2168.06 **(5)	0.131	0.90	0.90	0.120
Single factor E	9281.14	230	8254.38 ** (6)	0.230	0.70	0.69	0.170

Note: *N* = 751. ** *p* < 0.01. Three-factor model A combines perceived red tape and perceived overqualification into one factor. Three-factor model B combines the perceived overqualification and role overload into one factor. Three-factor model C combines the role overload and procrastination into one factor. Two-factor model D combines perceived red tape and perceived overqualification into one factor and role overload and procrastination into one factor. Single factor model E combines all the variables into one factor.

**Table 2:** Means, standard deviations, correlations, and reliability among the focal variables.

Variables	1	2	3	4	5	6	7	8	9	10	11	12
1.Gender	1											
2.Age	−0.093 *	1										
3.Tenure	−1.49 **	0.515 **	1									
4.Marital status	−0.53	0.390 **	0.381 **	1								
5.Education	−0.18	0.002	0.039	0.111 **	1							
6.Officer position	−1.47 **	0.223 **	0.270 **	0.174 **	0.281 **	1						
7.Type of work	0.122 **	−1.12 **	−1.76 **	−1.08 **	−1.15 **	−0.457 **	1					
8.Rank of organization	−0.57	0.033	−0.27	−0.32	−1.34 **	−1.17 **	0.161 **	1				
9.Red tape	−0.83 *	−0.02	0.016	0.018	0.025	0.067	−0.79 *	−0.053	(0.81)			
10.Role overload	−1.02 **	0.094 *	0.084 *	0.095 **	0.018	0.066	−1.25 **	0.002	0.460 **	(0.88)		
11.Overqualification	−0.46	0.020	0.007	−0.30	0.067	0.080 *	−0.76 *	−0.46	0.497 **	0.372 **	(0.89)	
12. Procrastination	−0.62	−0.76 *	−0.71	−0.58	−0.06	0.054	−0.72 *	0.025	0.341 **	0.250 **	0.457 **	(0.96)
Mean	0.46	32.51	1.43	1.68	2.07	1.60	1.79	2.07	3.73	3.80	3.51	2.82
SD	0.50	10.65	0.50	0.52	0.55	1.04	0.41	1.02	0.88	0.89	0.87	1.18

Note: *N* = 751. ** *p* < 0.01, * *p* < 0.05.

**Table 3:** Result of main effects and mediation effects.

Variables	Role Overload		Procrastination		
	Model 1	Model 2	Model 3	Model 4	Model 5
Intercept	4.008	2.112	3.956	2.039	1.711
Control variables					
Gender	−1.54	−0.88	−1.66	−0.99	−0.85
Age	0.003 *	0.004	−0.06	−0.04	−0.05
Tenure	0.036	0.038	−1.35	−1.33	−1.39
Marriage	0.093	0.091	−1.10	−1.13	−1.27
Education	−0.02	0.022	−0.04	0.020	0.016
Officer position	−0.11	−0.28	−0.08	−0.25	−0.21
Type of work	−2.31 *	−1.76 *	−2.88 *	−2.33	−2.06
Rank of organization	−0.02	0.018	0.017	0.037	0.034
Independent variables					
Perceived red tape		0.446 **		0.451 **	0.382 **
Mediating variable					
Role overload					0.156 **
*R* ^2^	0.02	0.213	0.014	0.125	0.134
∆*R*^2^		0.192		0.111	0.011
*F*	2.79	21.82	2.2	11.957	11.73

Note: *N* = 751. ** *p* < 0.01, * *p* < 0.05.

**Table 4:** Results of moderating effects.

Variables	Procrastination		
	Model 1	Model 2	Model 3
Intercept	3.956	3.807	3.718
Control variables			
Gender	−1.66	−1.04	−1.10
Age	−0.06	−0.08	−0.09 *
Tenure	−1.35	−1.34	−1.06
Marriage	−1.10	−0.42	−0.68
Education	−0.04	−0.49	−0.28
Officers position	−0.08	−0.16	−0.19
Type of work	−2.88 *	−2.11	−1.84
Rank of organization	0.017	0.039	0.044
Independent variables			
Role overload		0.122 *	0.152 **
Perceived overqualification		0.580 **	0.584 **
Interaction term			
RO X POQ			0.159 **
*R* ^2^	0.025	0.241	0.259
∆*R*^2^		0.216	0.018
*F*	2.2	21.64	21.6

Note: *N* = 751. ** *p* < 0.01, * *p* < 0.05.

**Table 5:** Results of the moderated indirect analysis.

		Indirect Effect	SE	95%CI
PO	−1SD	−0.17	0.024	−0.065, 0.028
	+1SD	0.108	0.035	0.040, 0.180
	Index of moderated mediation	0.070	0.018	0.038, 0.108

Note: N = 751. Bootstrap sample size = 5000.

## Data Availability

The data presented in this study are available on request from the corresponding author.

## References

[1] Metin U.B., Taris T.W., Peeters M.C. (2016). Measuring Procrastination at Work and Its Associated Workplace Aspects. Personal. Individ. Differ..

[2] Metin U.B., Taris T.W., Peeters M.C., Korpinen M. (2019). Validation of the Procrastination at Work Scale: A Seven-Language Study. Eur. J. Psychol. Assess..

[3] Steel P. (2007). The Nature of Procrastination: A Meta-Analytic and Theoretical Review of Quintessential Self-Regulatory Failure. Psychol. Bull..

[4] Svartdal F., Klingsieck K.B., Steel P., Gamst-Klaussen T. (2020). Measuring implemental delay in procrastination: Separating onset and sustained goal striving. Personal. Individ. Differ..

[5] Sharma S.K., Gupta J.N.D. (2004). Improving Workers’ Productivity and Reducing Internet Abuse. J. Comput. Inf. Syst..

[6] Sirois F.M. (2014). Procrastination and Stress: Exploring the Role of Self-Comparison. Self Identity.

[7] Tice D.M., Baumeister R.F. (1997). Longitudinal Study of Procrastination, Performance, Stress, and Health: The Costs and Benefits of Dawdling. Psychol. Sci..

[8] Van Eerder W. (2003). A Meta-Analytically Derived Nomological Network of Procrastination. Personal. Individ. Differ..

[9] Grund A., Fries S. (2018). Understanding Procrastination: A Motivational Approach. Personal. Individ. Differ..

[10] Lay C.H. (1992). Trait Procrastination and the Perception of Person-Task Characteristics. J. Soc. Behav. Personal..

[11] Zhou M. (2018). Gender Differences in Procrastination: The Role of Personality Traits. Curr. Psychol..

[12] De Amond S., Matthews R.A., Bunk J. (2014). Workload and Procrastination: The Role of Psychological Detachment and Fatigue. International. J. Stress Manag..

[13] Baldwin J.N. (1990). Perceptions of public versus private sector personnel and informal red tape: Their impact on motivation. Am. Rev. Public Adm..

[14] Bozeman B., Feeney M.K. (2011). Rules and Red Tape: A Prism for Public Administration Theory and Research.

[15] Bozeman B. (1993). A theory of government “red tape”. J. Public Adm. Res. Theory.

[16] Cooke D.K., Brant K.K., Woods J.M. (2019). The role of public service motivation in employee work engagement: A test of the job demands-resources model. Int. J. Public Adm..

[17] Kjeldsen A.M., Hansen J.R. (2018). Sector differences in the public service motivation- job satisfaction relationship: Exploring the role of organizational characteristics. Rev. Public Pers. Adm..

[18] Borst R.T. (2018). Comparing work engagement in people-changing and people-pro- cessing service providers: A mediation model with red tape, autonomy, dimensions of PSM, and performance. Public Pers. Manag..

[19] Hattke F., Vogel R., Znanewitz J. (2018). Satisfied with red tape? Leadership, civic duty, and career intention in the military. Public Manag. Rev..

[20] Taylor J. (2016). Working extra hours in the Australian public service: Organizational drivers and consequences. Rev. Public Pers. Adm..

[21] van den Bekerom P., Torenvlied R., Akkerman A. (2017). Constrained by red tape: How managerial networking moderates the effects of red tape on public service performance. Am. Rev. Public Adm..

[22] Hobfoll S.E. (1989). Conservation of Resources: A New Attempt at Conceptualizing Stress. Am. Psychol..

[23] Halbesleben J.R., Bowler W.M. (2007). Emotional exhaustion and job performance: The mediating role of motivation. J. Appl. Psychol..

[24] Bruning P.F., Campion M.A. (2018). A role–resource approach–avoidance model of job crafting: A multimethod integration and extension of job crafting theory. Acad. Manag. J..

[25] Halbesleben J.R., Harvey J., Bolino M.C. (2009). Too Engaged? A Conservation of Resources View of the Relationship between Work Engagement and Work Interference with Family. J. Appl. Psychol..

[26] Halbesleben J.R. (2006). Sources of Social Support and Burnout: A Meta-Analytic Test of the Conservation of Resources Model. J. Appl. Psychol..

[27] Hobfoll S.E., Halbesleben J.R., Neveu J.P., Westman M. (2018). Conservation of Resources in the Organizational Context: The Reality of Resources and Their Consequences. Annu. Rev. Organ. Psychol. Organ. Behav..

[28] Maynard D.C., Parfyonova N.M. (2013). Perceived overqualification and withdrawal behaviours: Examining the roles of job attitudes and work values. J. Occup. Organ. Psychol..

[29] Zhou X.G. (2011). Authoritative system and effective governance:The institutional logic of state governance in contemporary China. Open Era.

[30] Luksyte A., Spitzmueller C., Maynard D.C. (2011). Why do overqualified incumbents deviate? Examining multiple mediators. J. Occup. Health Psychol..

[31] Chen Y.Y., Zou Z.M., Pan J.H. (2017). Effects of overqualification on employees’ organizational citizenship behavior: From the perspective of emotion. Acta Pshcyology Sin..

[32] Chen X., Qi H., Wang X.Y., Huang X.M. (2019). The relationship between procrastination behavior and personality traits of college students: Mediating role of emotional regulation. Adv. Psychol..

[33] Ferrari J.R., Diaz-Morales J.F., O’Callaghian L., Diaz K., Argumedo D. (2007). Frequent Behavioral Delay Tendencies by Adults: International Prevalence Rates of Chronic Procrastination. J. Cross-Cult. Psychol..

[34] Blom R., Borst R.T., Voorn B. (2020). Pathology or inconvenience? A meta-analysis of the impact of red tape on people and organizations. Rev. Public Pers. Adm..

[35] Borst R.T., Kruyen P.M., Lako C.J., de Vries M.S. (2020). The attitudinal, behavioral, and performance outcomes of work engagement: A comparative meta-analysis across the public, semipublic, and private sector. Rev. Public Pers. Adm..

[36] Brunetto Y., Teo S.T.T., Farr-Wharton R., Shacklock K., Shriberg A. (2017). Individual and organizational support does it affect red tape, stress and work outcomes of police officers in the USA?. Pers. Rev..

[37] Lambert E.G., Hogan N.L., Jiang S. (2010). A preliminary examination of the relationship between organisational structure and emotional burnout among correctional staff. Howard J. Crim. Justice.

[38] Lin Y.Q. (2021). Constraints or activation:How red tape influence civil servants’ change-oriented behavior?. Public Adm. Rev..

[39] Bozeman B. (2012). Multidimensional red tape: A theory coda. Int. Public Manag. J..

[40] Quratulain S., Klan A.K. (2015). Red tape, resigned satisfaction, public service motivation, and negative employee attitudes and behaviors: Testing a model of moderated mediation. Rev. Public Pers. Adm..

[41] DeHart-Davis L., Pandey S.K. (2005). Red tape and public employees: Does perceived rule dysfunction alienate managers?. J. Public Adm. Res. Theory.

[42] Wang H.L., Zhang Q.J. (2016). The cost of feeling trusted: The study on the effects of feeling trusted from supervisor, role overload, job stress and emotional exhaustion. Manag. World.

[43] Rizzo J.R., House R.J., Lirtzman S.I. (1970). Role conflict and ambiguity in complex organizations. Adm. Sci. Q..

[44] George B., Pandey S.K., Steijn B., Decramer A., Audenaert M. (2021). Red Tape, Organizational Performance, and Employee Outcomes: Meta-analysis, Meta-regression, and Research Agenda. Public Adm. Rev..

[45] Giauque D., Anderfuhren-Biget S., Varone F. (2013). Stress Perception in Public Organisations: Expanding the Job Demands–Job Resources Model by Including Public Service Motivation. Rev. Public Pers. Adm..

[46] LePine J.A., LePine M.A., Jackson C.L. (2004). Challenge and Hindrance Stress: Relationships with Exhaustion, Motivation to Learn, and Learning Performance. J. Appl. Psychol..

[47] Eissa G., Lester S.W. (2017). Supervisor role overload and frustration as antecedents of abusive supervision: The moderating role of supervisor personality. J. Organ. Behav..

[48] Brislin R.W., Lonner W.J., Berry J.W. (1986). The wording and translation of research instruments. Field Methods in Cross-Cultural Research.

[49] Jacobsen C.B., Jakobsen M.L. (2018). Perceived organizational red tape and organizational performance in public services. Public Adm. Rev..

[50] Maynard D.C., Joseph T.A., Maynard A.M. (2006). Underemployment, job attitudes, and turnover intentions. J. Organ. Behav..

[51] Schaubroeck J., Cotton J.L., Jennings K.R. (1989). Antecedents and Consequences of Role Stress: A Covariance Structure Analysis. J. Organ. Behav..

[52] Beehr T.A., Walsh J.T., Taber T.D. (1976). Relationship of Stress to Individually and Organizationally Valued States: Higher Order Needs as a Moderator. J. Appl. Psychol..

[53] Tuckman B.W. (1991). The Development and Concurrent Validity of the Procrastination Scale. Educ. Psychol. Meas..

[54] Baron R.M., Kenny D.A. (1986). The moderator–mediator distinction in social psychological research: Conceptual, strategic, and statistical consideration. J. Personal. Soc. Psychol..

[55] Preacher K.J., Hayes A.F. (2004). SPSS and SAS procedures for estimating indirect effects in simple mediation models. Behav. Res. Methods Instrum. Comput..

[56] Hayes A.F. (2013). Introduction to Mediation, Moderation, and Conditional Process Analysis: A Regression-Based Approach.

[57] Harman H.H. (1976). Modern Factor Analysis.

[58] MacKinnon D.P., Fairchild A.J., Fritz M.S. (2007). Mediation analysis. Annu. Rev. Psychol..

[59] Hayes A.F. (2015). An index and test of linear moderated mediation. Multivar. Behav. Res..

[60] Edwards J.R., Lambert L.S. (2007). Methods for integrating moderation and mediation: A general analytical framework using moderated path analysis. Psychol. Methods.

[61] Scott P.G., Pandey S.K. (2005). Red tape and public service motivation:Findings from a National Survey of Managers in State Health and Human Services Agencies. Rev. Public Pers. Adm..

[62] Pandey S.K., Bretschneider S.I. (1997). The impact of red tape’s administrative delay on public organizations’ interest in new information technologies. J. Public Adm. Res. Theory.

[63] Moon M.J., Bretschneider S. (2002). Does the perception of red tape constrain it innovativeness in organizations? Unexpected results from a simultaneous equation model and implications. J. Public Adm. Res. Theory.

[64] Moynihan D.P., Pandey S.K. (2007). The role of organizations in fostering public service motivation. Public Adm. Rev..

[65] Mitchell M.S., Baer M.D., Ambrose M.L., Folger R., Palmer N.F. (2018). Cheating under Pressure: A Self-Protection Model of Workplace Cheating Behavior. J. Appl. Psychol..

[66] Maynard D.C., Taylor E.B., Hakel M.D., Chen O.T. (2009). Applicant overqualification: Perceptions, predictions, and policies of hiring managers. Organizational Behavior and Dynamics.

[67] Chen B., Zhou X., Guo G.X. (2021). Effect of perceived overqualification on emotional labor: A moderated mediation model. Nankai Rev..

[68] Wu C.H., Luksyte A., Parker S.K. (2015). Overqualification and subjective well-being at work: The moderating role of job autonomy and culture. Soc. Indic. Res..

[69] Bolino M.C., Feldman D.C. (2000). The antecedents and consequences of underemployment among expatriates. J. Organ. Behav..

[70] Hilgard E.R. (1980). The trilogy of mind: Cognition, affection, and conation. J. Hist. Behav. Sci..

[71] Wesse J., Bradley G.L., Hood M. (2019). Comparing Effects of Active and Passive Procrastination: A Field Study of Behavioral Delay. Personal. Individ. Differ..

